# Understanding Discussions of Sexual Assault in Young Women on a Peer Support Mental Health App: A Content Analysis

**DOI:** 10.1177/08862605211073112

**Published:** 2022-02-23

**Authors:** Joanna Collaton, Paula Barata, Stephen P. Lewis

**Affiliations:** 1 Department of Psychology, 3653 University of Guelph, Guelph, ON, Canada

**Keywords:** sexual assault, mental health, online communication, Me Too movement, youth

## Abstract

Trauma narratives may have been influenced by the Me Too movement, with thousands of individuals disclosing sexual violence stories online. Youth, the largest demographic of online users, may prefer the anonymity of the Internet to discuss experiences of sexual assault. Understanding the ways that young women, especially those experiencing mental health difficulties, discuss their experiences is important as they are at higher risk of revictimization and continued poor mental health. We searched for terms related to acts of sexual assault on a mental health peer-support app, TalkLife, and compared the number of posts during the initial wave of the Me Too movement (October 2017–March 2018) to the same time period in the previous year (October 2016–March 2017). We found a significant increase in posts related to sexual assault of 49.7% between the Pre and Post Me Too time periods (*p* < .001), controlling for a general increase in posts. A content analysis of 700 randomly selected posts found that a substantial number of young women used TalkLife to discuss their experiences of sexual assault, and these self-disclosures were mostly hopeless or depressing in tone. Additionally, neither the nature nor the number of self-disclosures varied across time points. The negative tone of the self-disclosures in the current study is worrying because the way women talk about their trauma can shape how they understand it, which could lead to negative self-appraisal and continued mental health difficulties. Online spaces have the potential to support young women and facilitate help-seeking, but we must be attentive to how they are used.

The Me Too movement has brought an unprecedented number of individual experiences of sexual assault to the attention of a broad audience. While first conceptualized by Tarana Burke in 2006, a tweet by actress Alyssa Milano initiated over 4.7 million Facebook posts and over 500,000 tweets with the hashtag #MeToo in October 2017 (Associated Press, 2017; Sayej, 2017). The Me Too movement became an international phenomenon, reaching at least 85 countries (Sayej, 2017), as survivors shared their personal experiences of sexual violence in conjunction with media exposés of powerful men. The Me Too movement has contributed to a cultural discourse on the implications of power, gender-based violence, and systemic silencing of survivors (Loney-Howes et al., 2021).

Approximately one in five young women and girls will be sexually assaulted in their lifetime (Finkelhor et al., 2014; Muehlenhard et al., 2017), and women are at higher risk compared to men. Sexual assault can include but is not limited to rape and child sexual abuse (Dube et al., 2005). Following sexual assault, women may experience anxiety, depression, posttraumatic stress, sexual problems, substance abuse, and suicidal thinking and behaviour (Briere & Jordan, 2004). As such, prevention and healing from acts of sexual violence are essential components of reducing the psychological burden of these experiences. An important part of being able to foster both prevention and healing is gaining a more fulsome understanding of how and where young women may talk about these experiences with others.

Discussing past experiences of sexual violence, especially within a therapeutic context, is an important part of growth and healing. Indeed, developing and defining a trauma narrative, a psychological approach to understanding one's traumatic experiences, is a key component of evidenced-based trauma treatment (Kaminer, 2006). Unfortunately, there are many barriers that prevent individuals, including youth, from accessing person-centred services as part of their recovery or healing goals (Gondek et al., 2017).

Many psychological services are cost-prohibitive, introducing a large systemic barrier that disproportionately impacts individuals from lower income brackets (Mojtabai, 2005; Vasiliadis et al., 2017). Beyond structural barriers to psychological services, many young people do not want to seek treatment due to stigma (Clement et al., 2015) or may not be ready to discuss intimate and sensitive details of their past in face-to-face contexts, especially with someone who is not of their age group. As such, peer-to-peer mental health support and the use of technology has been implicated as an innovative, relevant, accessible and cost-effective method for help-seeking, particularly in youth (Naslund et al., 2016). Indeed, the vast majority of teenagers and young adults have a smartphone and almost half indicate that they are continuously on their phones (Anderson & Jiang, 2018). They also report many positive outcomes of social media use, including feeling more connected and supported, indicating virtual sharing as an appealing avenue to healing for survivors of sexual assault (Anderson & Jiang, 2018).

Discussions of sexual assault occur across a variety of online and social media platforms (O’Neill, 2018). In this way, online disclosure may represent a means for trauma narrative synthesis as well as an emboldening and connecting opportunity for the many youth who have experienced sexual violence. Social media, specifically, is used for this purpose because it can serve as a space providing anonymity, safety, group identity and voice, especially for youth feeling isolated (Andalibi et al., 2016; O’Neill, 2018). Social media, on which much of the Me Too movement occurred, has been implicated as a space wherein women have disclosed experiences of sexual assault as well as discussed the prevalence of sexual violence and shared relevant news stories (Alaggia & Wang, 2020; Bogen et al., 2019; Manikonda et al., 2018). Being listened to and believed are highly important factors in better mental health adjustment after a sexually traumatic event (Ullman, 1996) and social media may provide youth this type of space. Additionally, virtual communication may allow for more control over individual narratives via thoughtful choice of language increasing feelings of safety. We do not know, however, the role of the Me Too movement among young women who live with mental health challenges, highlighting an important area of further exploration.

The Internet poses an interesting dichotomy as survivors of sexual violence may be afforded anonymity and connection with others while also providing responders the same anonymity to respond negatively. These responses may put youth at risk of revictimization (Filipas & Ullman, 2001) by contributing to harm related to not being believed, experiences of shame (McElvaney et al., 2014), as well as fomenting mental health difficulties (Mitchell et al., 2001, 2007). Negative reactions to disclosures can also worsen mental health (Ullman & Peter-Hagene, 2014). Thus, it is difficult to determine in what instances survivors may deem an online space to be ‘safe’ to disclose their personal experiences. By understanding the nature of online posts about sexual assault, we may be better positioned to identify the needs of youth with mental health difficulties or gain insight into the role of social media in these contexts. Previous research points to online spaces such as Twitter and Reddit as places for women to share disclosures of sexual assault and to share news stories of sexual violence (Alaggia & Wang, 2020; Bogen et al., 2019; Manikonda et al., 2018; Modrek & Chakalov, 2019; O’Neill, 2018). However, we do not know how young women with mental health challenges who are more likely to have a history of sexual assault (Chen et al., 2010) discuss their experiences of sexual assault online. Theoretically, the way that young women discuss their experiences may have changed in the context of the Me Too movement. For example, Alaggia and Wang (2020) posit that survivors may have experienced resilience and connection via online disclosures of sexual assault in the context of the Me Too movement. As such, participating in and viewing fellow disclosures from similar-aged peers may result in feelings of empowerment and strength. Given the recency of the Me Too phenomenon (October 2017), it remains unclear how youth, especially youth with mental health challenges, are discussing their experiences of sexual assault and if those discussions have changed in the context of the movement.

## Current Study

Following the above, the present study examined social media posts on TalkLife, a mental health peer-support app targeted to teenagers and young adults experiencing mental health difficulties. As a first step, we compared the quantity of posts on the app relating to sexual assault within the first 6 months after the initial wave of Me Too with posts made during the same time period in the previous year. Since Me Too has increased online sexual assault disclosures in general, we hypothesized that discussions relating to sexual assault would increase over time and that the tone of such discussions would be increasingly positive in valence (e.g. more likely to be associated with moods such as *happy, inspired *and* positive*) in the second time period. Next, and to provide more nuance and context to the retrieved posts, a random subset of posts relating to self-disclosures from both time points were content analysed and are described in detail. We hypothesized an increase in self-disclosures during the initial wave of the Me Too movement and that these disclosures would be more likely to be paired with empowering language (e.g. survivor vs. victim).

## Method

### TalkLife

TalkLife is a free, peer support mobile app for youth experiencing mental health difficulties available in 120 countries. At the time of data collection, there were over 600,000 TalkLife users. TalkLife users post content including details of recent events, personal experiences and stories of mental health struggles; users can also comment on other posts.

### Search Strategy and Data Handling

#### Quantitative Search

To retrieve posts for the quantitative analysis, a search for TalkLife posts related to sexual assault was conducted in the initial wave of the Me Too movement (4 October 2017–1 April 2018) as well as the same time period in the previous year (4 October 2016–1 April 2017). In doing so, all posts with 1–32,767 characters (i.e. the maximum number of characters allowed per cell in MS Excel) were exported and retained. To conduct the search, terms selected from relevant literature (e.g. systematic review search terms) were used to identify potentially relevant original posts (i.e. we did not look at comments on posts); search strategy and terms are in Supplementary Appendix A.

#### Content Analysis Search

To retrieve posts for the content analysis, we used the same search terms used for the quantitative search and added the search terms ‘victim’, ‘survivor’, ‘#MeToo’, as well as several prominent names of individuals associated with the movement given their salience to the research questions (Supplementary Appendix A). These additional terms were not used in the quantitative analysis as ‘victim’ and ‘survivor’ are used in other contexts (e.g. mental health) which would therefore introduce noise into the search which we were unable to audit. We searched the same time period as the quantitative analysis (i.e. 4 October 2016–1 April 2017 and 4 October 2017–1 April 2018). All posts were retained.

Users of TalkLife pair their posts with a ‘mood’ by choosing one of 44 pre-determined options available on TalkLife (e.g. ‘*encouraged*’ and ‘*anxious*’). For the purposes of the current study, these were trichotomized into positive valence, negative valence, or neither, to identify whether there was a change in overall mood valence across the two time points. To account for interrater reliability when categorising moods, two members of the research team sorted the moods (see Supplementary Appendix B). Raters had excellent agreement (90.5–97.6% agreement); discrepancies were discussed by the research team to reach consensus. The final data file therefore included the text from the post, the date, tagged mood valence, number of comments and views on each post, as well as available user demographic data.

Prior to study commencement, TalkLife granted access to the data for use in research. This is in keeping with TalkLife’s privacy policies, wherein it is noted that data may be used for research purposes. In addition, ethical clearance was granted to the research team prior to study commencement by our Institutional Research Ethics Board.

### Quantitative Analysis

Post characteristics (age, number of views and number of comments) were tabulated for the sample as a whole and then for both prior to (i.e. Time 1) and during the Me Too movement (i.e. Time 2). Number of comments is auto-calculated by TalkLife; the comment must have at least one character to be counted. We assessed for group differences across time points via t-tests.

We first looked at the overall discussion related to acts of sexual violence by aggregating all search terms over time (i.e. number of sexual assault posts at Time 1 compared to Time 2). We compared the exact same search terms over time (see Supplementary Appendix B). If a resultant post had more than one of the search terms (e.g. the same post included both ‘rape’ and ‘sexual assault’), it was only included once in the aggregated data. Comments on posts were not included in the analysis. Given that the number of total posts on the app increased considerably (by 69.1%) from Time 1 to Time 2, we used weighted chi-square tests to assess for significant differences between the number of posts to account for this overall increase (i.e. by 69.1%). An odds ratio was calculated for the number of sexual assault posts across time points. The same analysis (chi-square test and odds ratio) was completed for mood of post over time (i.e. comparing positive- and negative-valence mood).

### Content Analysis

To complement the above analysis and to better understand the nature of TalkLife posts related to sexual assault, a mixed inductive-deductive content analysis was completed using data from both time points (Hsieh & Shannon, 2005). To create a coding rubric, we randomly selected 50 posts (25 from Time 1, 25 from Time 2) to review; posts were identified using the above content analysis search strategy. Codes were informed by these posts and extant literature (Bogen et al., 2019) and were included in line with pre-established research questions. This resulted in a working version of the coding rubric which was finalised following discussion, modification, clarification, and addition of variables in team meetings. The coding team, composed of two white, cis-women under 30, conducted two rounds of practice coding with 28 posts (half from each time point) until percent agreement for all variables was above 70% and coders reported comfortability with the rubric. In total, 10 superordinate variables and 37 subordinate variables were included as part of the final rubric. Posts were excluded if they were too vague to code (e.g. if a poster detailed ‘abuse’ without clarifying it was sexual abuse) or if they were non-English.

**Figure 1.**
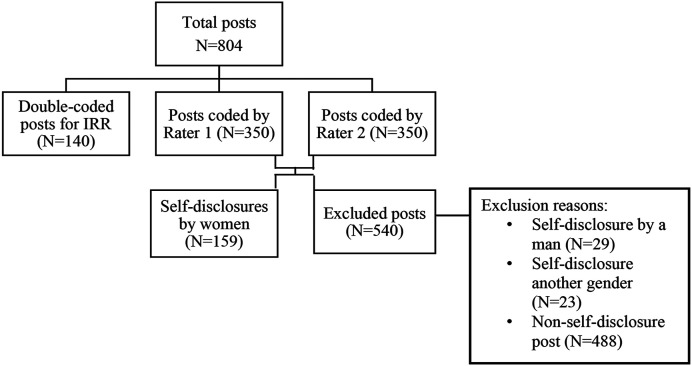
Flow diagram of post inclusion/exclusion process.

The data included for this study were collected as part of a larger project on TalkLife discussions of sexual violence and the feminist movement and as such, only a subset of collected and coded posts are presented in this paper. For the larger study, we randomly selected 840 posts. Two coders coded the same 140 posts (20% of posts, keeping with established norms from prior research e.g. Hinduja & Patchin, 2008; Webb et al., 2017) and 350 posts individually (175 pre, 175 post). The double-coded posts were excluded from the final analysis. For the present analysis, we only examined self-disclosure of sexual assault style posts by women (*n*=159 posts) and excluded posts that did not include a sexual assault disclosure (*n*=488 posts) or were disclosures by men (*n*=29 posts) or individuals who did not disclose a gender of man or woman (*n*=23 posts). See Figure 1 for a flow diagram of included posts.

### Variables coded

#### Self-Disclosure

We coded for the presence of a disclosure of sexual assault. We also coded whether the event happened within the past year, the word(s) used to describe the act (e.g. rape, assault, violate and forced), who perpetrated the act (e.g. family member and partner), the gender of the perpetrator, whether the poster described themselves as a victim or survivor, and whether they explicitly mentioned telling other people about the incident.

#### Tone of the Post

To better understand the overall tone of the post, we coded tone as either positive (i.e. empowering or hopeful), negative (i.e. hopeless or depressing), neutral (i.e. devoid of emotion-laden language) or mixed (i.e. included both positive and negative language).

#### Mental Health Symptom or Label

We coded for the presence of symptoms or explicit mention of depression, anxiety, posttraumatic stress disorder (PTSD), suicidal thinking or behaviour or other mental health difficulties (e.g. disordered eating). Similarly, negative self-talk, including self-deprecatory language or negative language about the self, was coded.

#### Rape Myths

We coded for the presence of rape myths, including posters endorsing rape myths related to their own experience (e.g. that they did not say no and therefore it was their fault) as well as if they posted about an experience where someone endorsed a rape myth to them (e.g. their parent suggested that they should not have been drinking). We also coded separately for posts that debunked rape myths. We coded for 11 common rape myths that blame victims (e.g. women ask for it in various ways), exonerate perpetrators (e.g. male aggression is normal), or make some assault invisible (e.g. acquaintance assault). We also included an ‘other’ rape myth option for posts that clearly support taking away blame from the perpetrator and shaming the survivor but did not fit the other options. Rape myths aligned with the subscales of the Illinois Rape Myth Acceptance Scale (e.g. she asked for it; it was not really rape; she lied; he did not mean to; Payne et al., 1999). We also added several other rape myths to code based on investigator knowledge, including that men cannot be raped and that survivors will have a good memory of the event.

#### Help and Information-Seeking

Given the nature of the mobile app as a peer support mental health app, we coded whether posters were explicitly seeking and soliciting help on the app. We also coded whether posters were seeking information about an experience, definition or similar.

### Interrater reliability

To assess interrater reliability, percent agreement and Gwet’s AC1, an agreement coefficient, was calculated. Gwet’s AC1 was chosen over kappa statistics due to the volatility of kappa; specifically, high percent agreement of variables can result in paradoxically low kappa, thus misrepresenting the data (Gwet, 2008; Quarfoot & Levine, 2017). Percent agreement and AC1 were calculated using ReCal and RStudio Version 1.1.456 with the ‘rel’ package, respectively (Freelon, 2010; LoMartire, 2020). Percent agreement ranged from 82.7 to 100% with good to excellent agreement (AC1 = .64–.99) (Landis & Koch, 1977). See Supplementary Appendix B for interrater reliability agreement indicators.

### Frequencies and Cross-Tabulation

Like the above analysis, we calculated age of poster across time point with a t-test to assess for differences. As one of the primary research questions was to better understand the nature of self-disclosures, we conducted crosstabulations with disclosures for the following variables for both Time 1 and Time 2: associated mood valence, tone, mental health label, negative self-talk, use of rape myth, help and information seeking. Frequencies and percentages were computed to understand the presence of collected variables of interest. Chi-square tests were completed to assess for the differences over time for the number of disclosures, as well as help- and information-seeking. We hypothesized that disclosures would increase in Time 2 and that they would be associated with more positive and empowering language (measured by whether they described themselves as a survivor as well as the tone of the post). We also compared the presence of rape myths and negative self-talk over time within the context of self-disclosures. As we did not know how prevalent help- and information-seeking, rape myths and negative self-talk would be on the app, we did not have a priori hypotheses about changes over time. SPSS Version 26 was used for this analysis.

## Results

### Demographic and Post Characteristics

Overall, 3765 posts were retrieved using the search criteria (1181 from Time 1, 2584 from Time 2). The mean age of posters was 19.0 years (SD = 4.7). Age of the sample differed marginally, although significantly, between time points, with mean age of Pre Me Too posters as 18.1 (SD = 3.7) and mean age of Time 2 posters as 19.5 (SD = 5.0; *p* < .001, *n* = 265 missing). Views and comments were more common at Time 2 (*p* < .001) with an average of 40.3 views (SD = 31.6) at Time 1 and 63.0 (SD = 96.8) at Time 2; there were 4.2 comments per post (SD = 5.7) at Time 1 and 5.2 (SD = 10.5) in Time 2 (*p* < .001).

### Quantitative Analysis

As hypothesized, posts with discussions relating to sexual assault increased over time by 118.8%; this is 49.7 percentage points higher than expected, given the 69.1% increase in the total number of posts across time points, *χ*^2^ (1, *N* = 1,462,128) = 53.92, *p* < .001. There was a small but significant effect size, such that the odds were 1.29 times higher that someone posted about sexual assault in Time 2 compared to Time 1 (OR = 1.29, 95% CI: 1.21, 1.39). Posts were more likely to be paired with a negative mood (*n* = 875, 75.5% at Time 1, *n* = 1998, 78.1% at Time 2) versus a positive mood (*n* = 172, 14.8% at Time 1, *n* = 322, 12.6% at Time 2), although this did not differ significantly over time *χ*^2^ (1, *N* = 3367) = 3.74, *p* = .053. The odds of a post being paired with a negative mood Post Me Too was 1.22 times likelier compared to Pre Me Too (OR = 1.22, 95% CI: 0.99, 1.49). See Table 1 for a summary of post characteristics.

**Table 1.** Post Characteristics for Quantitative and Content Analysis.

Analysis	Variable	Pre Me Too (Time 1)	Post Me Too (Time 2)	Overall Sample	*p*-value
Quantitative	Number of posts, *n*(%)	1181 (32.1)	2584 (68.6)	3765	<.001
	Mood				
	Negative, *n*(%)	875 (75.5)	1998 (78.1)	2873	.053
	Positive, n(%)	172 (14.8)	322 (12.6)	494	
	Age, mean (SD)	18.1 (3.7)	19.5 (5.0)	18.8 (4.5)	<.001
	Views, mean (SD)	40.3 (31.6)	63.0 (96.8)	55.1 (74.5)	<.001
	Comments, mean (SD)	4.2 (5.7)	5.2 (10.5)	4.6 (7.8)	<.001
Content	Number of posts, *n*(%)	83 (52.2)	76 (47.8)	159	.616
	Mood				
	Negative, *n*(%)	68 (81.9)	64 (84.2)	132	NC
	Positive, n(%)	4 (4.8)	2 (2.6)	6	
	Age, mean (SD)^a^	17.8 (4.3)	18.9 (5.2)	18.4 (4.7)	.161
	Views, mean (SD)	42.3 (29.2)	69.6 (95.6)	52.6 (62.9)	.019
	Comments, mean (SD)	5.0 (6.3)	5.8 (8.9)	5.1 (7.0)	.558

*Note.* NC = not calculable due to low cell count.

^a^*n* = 1 missing

### Content analysis

From the 700 randomly selected posts (350 from each time period and excluding 140 double-coded posts for reliability assessment), 159 were of women’s self-disclosures and thus included for this analysis. Table 1 summarizes post characteristics.

There were 159 disclosures overall, 83 (52.2%) at Time 1 and 76 (47.8%) at Time 2 (Table 2). Contrary to the hypothesis, there was not a significant difference between number of disclosures at Time 1 compared to Time 2 (*χ*^2^ (1, *N* = 259) = .080, *p* = .778). Twenty-five posts (15.7%) included multiple disclosures of sexual assault (ranging between two to 10), and 35 (22.0%) posts indicated that the event occurred in the past year.

**Table 2.** Self-Disclosure Characteristics.

Superordinate Category	Subordinate Variable	Pre *n*(%)	Post *n*(%)	Total *n*(%)	*p*-value
Self-disclosure	Present	83	76	159	.080
	Past year	23 (27.7)	12 (16.0)	35 (22.2)	
	Perp is family	8 (9.6)	13 (17.3)	21 (13.3)	
	Perp is a friend	6 (7.2)	5 (6.6)	11 (7.0)	
	Perp is a partner	14 (16.9)	10 (13.3)	24 (15.2)	
	Perp is a man	36 (43.3)	37 (49.3)	73 (46.2)	
	Perp is a woman	1 (1.2)	0 (0)	1 (0.6)	
	Label: Rape	50 (60.2)	38 (50.7)	88 (55.7)	
	Label: Assault	4 (4.8)	8 (10.7)	12 (7.6)	
	Label: Sexual violence	2 (2.4)	1 (1.3)	3 (1.9)	
	Label: Violate	0 (0)	0 (0)	0 (0)	
	Label: Touched	6 (7.2)	5 (6.6)	11 (7.0)	
	Label: Forced	3 (3.6)	1 (1.3)	4 (2.5)	
	Label: Harassment	4 (4.8)	0 (0)	4 (2.5)	
	Label: Catcall	1 (1.2)	0 (0)	1 (0.6)	
	Label: Molest	12 (14.5)	11 (14.6)	23 (14.6)	
	Label: Coerce	5 (6.0)	0 (0)	5 (3.2)	
	Label: Advantage of	2 (2.4)	1 (1.3)	3 (1.9)	
	Label: Tech-mediated	3 (3.6)	2 (2.7)	5 (3.2)	
	Label: Sexual abuse	5 (6.0)	8 (10.7)	13 (8.2)	
	Label: Other non-consensual act	3 (3.6)	1 (1.3)	4 (2.5)	
	Label: Kiss	2 (2.4)	3 (4.0)	5 (3.2)	
	Label: Rape threats	2 (2.4)	1 (1.3)	3 (1.9)	
	Label: Photos taken	2 (2.4)	0 (0)	2 (1.3)	
	Self-describe: Victim	3 (3.6)	4 (5.3)	7 (4.4)	
	Self-describe: Survivor	1 (1.2)	0 (0)	1 (0.6)	
	Told others	14 (16.9)	11 (14.7)	25 (15.8)	
Mental health	Any MH symptom	35 (42.2)	38 (50.7)	73 (46.2)	
	Depression	15 (18.1)	15 (20.0)	30 (19.0)	
	Anxiety	9 (10.8)	7 (9.3)	16 (10.1)	
	PTSD	5 (6.0)	7 (9.3)	12 (7.6)	
	Suicidal thinking	12 (14.5)	13 (17.3)	25 (15.8)	
	Other symptom	7 (8.4)	8 (10.7)	15 (9.5)	
	Negative self-talk	21 (25.3)	23 (30.7)	44 (27.8)	
Rape myths	No means yes	2 (2.4)	2 (2.7)	4 (2.5)	
	Women are asking for it	4 (4.8)	1 (1.3)	5 (3.2)	
	Boys are naturally aggressive and do not know better	0 (0)	0 (0)	0 (0)	
	Alcohol leads to aggression	0 (0)	0 (0)	0 (0)	
	A conviction ruin life/good guys who make mistakes should not be greatly punished	1 (1.2)	0 (0)	1 (0.6)	
	Women are lying	4 (4.8)	2 (2.7)	6 (3.8)	
	Men cannot be victims	0 (0)	0 (0)	0 (0)	
	Rape is usually committed by strangers	2 (2.4)	1 (1.3)	3 (1.9)	
	Survivors can prevent assault (e.g. by saying no, running away, closing their legs)	3 (3.6)	2 (2.7)	5 (3.2)	
	Survivors will have a good memory	0 (0)	0 (0)	0 (0)	
	Abused abuse people	0 (0)	1 (1.3)	1 (0.6)	
	Rape is a joke or not a big deal	1 (1.2)	1 (1.3)	2 (1.3)	
	Reporting is an easy process	0 (0)	1 (1.3)	1 (0.6)	
	Young people cannot be raped	0 (0)	1 (1.3)	1 (0.6)	
	Men deserve nude photographs	1 (1.2)	0 (0)	1 (0.6)	
Information-sharing	Dispelling rape myth	5 (6.0)	7 (9.3)	12 (7.6)	
Help-seeking		7 (8.4)	10 (13.3)	17 (10.8)	.321
Information seeking		6 (7.2)	6 (8.0)	12 (7.6)	.855
Overall tone	Positive	3 (3.6)	6 (8.0)	9 (5.7)	
	Negative	64 (77.1)	53 (70.7)	117 (74.1)	
	Neutral	10 (12.0)	11 (14.7)	21 (13.3)	
	Mixed	6 (7.2)	5 (6.7)	11 (7.0)	

With respect to relationship with the perpetrator, many posters did not include this information (*n* = 91, 57.2%). When they did, 24 were current or past partners (15.1%), 21 were family members (13.2%), and 11 were friends or schoolmates (6.9%). Similarly, approximately half of posts did not include the perpetrators’ gender (*n* = 84, 52.8%). When gender of the perpetrator was listed, it was most commonly men (*n* = 73, 45.9% of all posts).

The most common terms used to describe the act were rape (*n* = 88, 55.3%) and molest (*n* = 23, 14.4%). Very few posters described themselves as either a victim (*n* = 7, 4.4%) or survivor (*n* = 1, 0.6%); low endorsement of these terms precluded pre-post Me Too analysis. Twenty-five posters indicated that they had told someone else about the assault (*n* = 25, 15.7%). Many posts described a mental health difficulty in the same post as a self-disclosure (*n* = 74, 46.5%). Negative self-talk was included in 44 (27.8%) posts.

Rape myths were coded in one-fifth of self-disclosures (*n* = 29, 18.2%; *n* = 18 at Time 1, *n* = 11 at Time 2, *χ*^2^ (1, *N* = 159) = 1.30, *p* = .239); this included posts in which users were either endorsing a rape myth or described an experience when someone else endorsed a rape myth. There were five rape myths that were coded and labelled post hoc, meaning they were not in the coding rubric; this included that rape is a joke, young people cannot be raped, men deserve nude photographs if women have shared other (non-sexual) photos online, and individuals who have been sexually abused will abuse others. Four women also discussed having experienced slut-shaming. In comparison, 12 posts (7.5%) made an attempt to dispel a rape myth (e.g. stating that it is never a woman’s fault).

A number of posts were explicitly seeking help (*n* = 17, 10.7%) or information (*n* = 12, 7.5%) related to the disclosure; neither of these differed significantly between time points (*χ*^2^ (1, *N* = 158) = .985, *p* = .321 for help-seeking, (*χ*^2^ (1, *N* = 158) = .033, *p* = .855 for information-seeking). The majority of self-disclosures had a negative tone (*n* = 118, 74%). There were slightly fewer posts with a negative tone in Time 2, although this was not significantly different (*χ*^2^ (1, *N* = 159) = .760, *p*=.383).

## Discussion

The way that individuals discuss sexually traumatic events may inform their understanding and appraisal of such events (Mendes et al., 2019) which has implications for their post-assault mental health. Understanding the ways that young women discuss their experiences of sexual assault is important, because the labels that researchers, clinicians, and law enforcement ascribe to individuals’ experiences does not always align with their conceptualization of their experience. We found significant increases in discussions of sexual assault pursuant to the Me Too movement on the app; however, we did not find an actual increase in disclosures over time. A closer look at the data show that girls and young women disclosed personal details related to stories of rape, molestation and other forms of assault on the app and overwhelmingly paired these stories with negative-valence moods, including how they elicit sadness, heartbreak, fear and insecurity. They typically did not self-describe as a victim or a survivor of their experience within their posts, contrary to our hypothesis that youth would pair their disclosures with empowering language (i.e. survivor language) during the Me Too movement time period. Within disclosures, users sometimes espoused rape myths in that they either personally reported them or suggested that others had endorsed them in relation to their experience which may put them at risk of poor mental health (Campbell et al., 2009).

In contrast to other social media sites on which users provide details about their assault (Bogen et al., 2019), TalkLife users tended to be more private about such information, typically not disclosing their relationship to the perpetrator but rather labelling the act (e.g. rape) and pairing it with a discussion of their mental health; this was observed in half of the disclosures. As such, TalkLife users may be more likely than other social media users to connect their sexual assault to their mental health difficulties. Given the nature and focus of the app, it is unsurprising that TalkLife users are discussing mental health more than on other social media.

This study was able to tap into a vulnerable, difficult-to-reach population, namely young survivors of sexual violence who are using a peer-support platform in support of their mental health. The vulnerability of the sample is underscored by their age, the experiences they reported, and their probable mental health status. For example, younger age, like those in this study (M_age_ = 19 years), has been shown to predict PTSD and depression symptoms following a sexual trauma (Elliott et al., 2004) thus highlighting the vulnerability of this sample. Within disclosures, TalkLife users from this study were more likely to describe instances of rape and molestation, which are often more violent than other acts of sexual violence (e.g. sexual harassment). This is concerning given research indicating that a higher degree of violence is associated with worse post-assault mental health (Cascardi et al., 1996; Halligan et al., 2003). Rape and molestation are also the most likely forms of sexual violence to be related to PTSD among women (Kessler et al., 1995). We also found that 16% of disclosures indicated multiple incidents of sexual violence. As cumulative trauma predicts depression, anxiety and PTSD (Campbell et al., 2009), this is likely a consideration in the mental health of these survivors. As such, we were able to identify a high-need group of young women who may be vulnerable to pervasive mental health difficulties and learn more about their online behaviour.

### Limitations and Future Directions

Limitations of the study include the observational design, limited demographic and geographic data and assumptions employed to handle a large amount of data. Given the design, we were not able to follow-up with participants to explore the mental health and trauma experiences of young women who disclose sexual violence online. Follow-up studies should better ascertain the nature of their experiences and the subsequent impact on their mental health. While this study was able to gather a large amount of observational data, further inquiry into the impact of posting online about experiences of sexual trauma are warranted. Indeed, it would also be important to understand the motivations behind posting to identify potential solutions to unmet needs in this population.

We also had limited demographic and geographic information, precluding an intersectional lens. The majority of high-profile celebrities lauded for their bravery of sharing their sexual assault stories were wealthy, white, able-bodied, cis women in North America. As such, many girls and women who do not fall into this narrow demographic group may not see themselves in these public narratives and thus may remain silenced by systemic and oppressive barriers to justice after sexual assault. Hence, future research ought to further explore how the Me Too movement has impacted, if at all, girls and women not fitting this dominant group. In addition, the sociodemographic factors of the coding team (i.e. white women under 30) were generally aligned with one another and with the many women who were more likely to share their stories in the context of the Me Too movement. A more diverse research team may have brought different insights into the content analysis and should be sought out for future, similar studies. Privacy limitations also meant that we could not access the IP addresses of users, meaning regional-based analyses were prohibited. Examining regional differences is an important future direction to consider.

To assess a large amount of data (almost 7000 posts), we dichotomized the majority of moods (i.e. tagged moods such as *sad*, *anxious* and *excited* on each post) into either a negative or positive valence. While this method allowed us to make comparisons at a high-level, such that most posters were pairing their post with a negative-valence mood, this process ignored the nuance and context of posts. For example, anger and frustration are normative and potentially helpful emotions to experience after a sexual assault (Smith & Kelly, 2001). To mitigate this limitation, we more thoroughly explored a random subset of the data and found that, in fact, many of the posts were deemed hopeless and depressing in nature and few posters used empowering language.

The quantitative comparison was conducted on all the posts using the same search terms for both time periods; however, we added search terms that were specific to Me Too before randomly selecting 350 posts from each time period for the content analysis. This was done to ensure we had a sufficient sample of posts that mentioned the Me Too movement for the broader study. This decision may have impacted our comparison of disclosures across the two time periods. For example, there may have been more non-disclosure posts that mentioned Me Too in the sample of the second time period. Nevertheless, this should not have impacted our analysis of the actual disclosures that were carefully examined from both time periods.

These challenges notwithstanding, the study findings indicate that young women who use TalkLife are both expressing their experiences and potentially being exposed to other negative-laden disclosures and larger discussions of sexual assault (the average view of self-disclosure posts was 55.4 per post). Given the number of views of each post, it is also important to consider that this may be a particularly vulnerable group of youth who are experiencing significant mental health difficulties and, as such, they may be more vulnerable to reports of others’ trauma; this is the case for social media use in individuals who self-injure and those with eating disorders (Campaioli et al., 2017; Lewis & Seko, 2016). Future studies may explore the impact of viewing negative-laden descriptors of sexually traumatic events.

We also did not analyse the comments to disclosure posts. While using online communities can be empowering for survivors of sexual assault (O’Neill, 2018), these benefits may be due to positive or supportive interactions with other victim-survivors which may not be the majority of TalkLife users. Of the self-disclosures on TalkLife examined in this study, 13% had no comments with the majority of these self-disclosure posts having fewer than four replies. Given the negative implications of inflammatory or invalidating responses to disclosures of sexual assault (Filipas & Ullman, 2001), understanding the content of comments on these types of posts is a worthwhile avenue for future research.

### Implications

Narrative style posts, like the kinds that included disclosures, may serve to develop and maintain parts of self-concept. Of concern, it seems that these narratives are mostly being shared in conjunction with negative-laden affect and a hopeless tone; 86% of the disclosures were considered hopeless or depressing in our content analysis. In addition, 28% of disclosures included negative self-talk. This is particularly concerning given that negative self-appraisals maintain PTSD symptoms (Halligan et al., 2003) and are related to emotion regulation difficulties and low self-compassion (Barlow et al., 2017).

The prevalence of rape myths within disclosures is also concerning. While we do not yet know the impact of the Me Too movement on the public understanding of rape myths or related knowledge of sexual assault (e.g. the definition of consent, prevalence of sexual violence), many high-profile cases saw power being stripped from long-time abusers including several convictions (Chicago Tribune, 2019). At least in this sample, the prevalence of rape myths, seen in almost 20% of disclosures with no significant reductions over time, suggests that it remains a barrier to healing, reporting, and justice for survivors (Prochuk, 2018). This has implications for this population including continued poor mental health, self-silencing, self-blame, shame and stigma (Kennedy & Prock, 2018). Of note, rape myths may be becoming more subtle over time (O’Connor et al., 2018); this may have been reflected in the rape myths we identified post hoc which may be useful for future research.

TalkLife and similar peer support or social media apps may benefit from better integration of psychoeducational and therapeutic resources. Given the prevalence of sexual assault in populations of individuals with mental health challenges, TalkLife and similar platforms may be used to support and educate users beyond peer support. This may include more direct and personalized resources to rape and sexual health clinics depending on IP address, as has been done with other mobile apps (e.g. for suicidal ideation in postpartum depression, Collaton et al., 2022). For peers responding to these types of disclosures, consent education and psychoeducation on the best ways to support someone after a disclosure is recommended, given the importance of social reactions in determining post-assault mental health and post-assault recovery (Gueta et al., 2020; Ullman, 2004; Ullman & Peter-Hagene, 2014).

Clinical implications include the need for regular social media assessment to identify the frequency and purpose of social media use among youth. Specifically, it is important to consider the social network of clients as well as on- and offline reactions to disclosures that they have received (Ullman & Peter-Hagene, 2014). However, a client’s support system and social network is only one factor to consider. Developing a trauma narrative within a therapeutic space is common to many trauma-based therapies (Kaminer, 2006); without formal supports and in the face of negative-laden narratives from peers, young women may be at risk of reifying their trauma and developing hopelessness for recovery. As such, assessing the ways in which this narrative has been established and addressing the related negative-laden cognitions is likely an important therapeutic target.

Overall, we were able to reach and identify discussions from a vulnerable group of young survivors of sexual assault who are seeking peer support. As some of these users may not participate in research studies, reach the therapy room, nor seek other forms of support or justice, this methodology represents a novel measure of their experiences with sexual violence and mental health. Apps such as TalkLife may represent a key intervention point between a disclosure and the recovery process after sexual assault as well as a forum to connect young women to share these experiences.

## Supplemental Material

sj-pdf-1-jiv-10.1177_08862605211073112 – Supplemental Material for Understanding Discussions of Sexual Assault in Young Women on a Peer Support Mental Health App: A Content Analysis Click here for additional data file. Supplemental Material, sj-pdf-1-jiv-10.1177_08862605211073112 for Understanding Discussions of Sexual Assault in Young Women on a Peer Support Mental Health App: A Content Analysis by Joanna Collaton, Paula Barata and Stephen P. Lewis in Journal of Interpersonal Violence
