# Supplementary material for: Understanding Discussions of Sexual Assault in Young Women on a Peer Support Mental Health App: A Content Analysis
Source: J Interpers Violence. 2022 Feb 23;37(23-24):NP22811–33. doi: 10.1177/08862605211073112 (PMC9679558; doi:10.1177/08862605211073112)
Supplement: sj-pdf-1-jiv-10.1177_08862605211073112 – Supplemental Material for Understanding Discussions of Sexual Assault in Young Women on a Peer Support Mental Health App: A Content Analysis [file sj-pdf-1-jiv-10.1177_08862605211073112.pdf]

APPENDIX A: Search Strategy

Part 1 Search terms for the quantitative analysis:

rape
sexual violence
sexual assault
molest
sexual harassment
sexual abuse
street harassment
catcall
sexual aggression
sexual coercion
domestic violence
dating violence
exploit
forced sex
touched me
advantage of me
abuser

Part 2 Search terms:

For the content analysis, search terms between October 2017 – Apr 2018 were the same with these additional terms:
Victim
Survivor
Harvey Weinstein
#MeToo
#me too
Rose McGowan
Alyssa Milano
Kevin Spacey
Bill Cosby
Ashley Judd
McKayla Maroney
Louis C.K
James Franco
Donald Trump
Larry Nassar
R. Kelly

APPENDIX B: Supplemental Tables

Supplemental Table 1: User-tagged moods by valence

**Positive Mood**	**Negative Mood**
Amazed	Afraid
Amused	Angry
Calm	Annoyed
Caring	Anxious
Encouraged	Embarrassed
Excited	Exhausted
Happy	Frustrated
Inspired	Furious
Loving	Heartbroken
Playful	Insecure
Positive	Irritated
Proud	Jealous
Relaxed	Lonely
Relieved	Nervous
Supportive	Numb
	Sad
	Shocked
	Sick
	Stressed
	Tired
	Worried

*Other TalkLife moods not included: Astonished, Confused, Hungry, Meh, Surprised

Supplemental Table 2: Interrater reliability agreement

Variable Name	Percent agreement	AC1
Not Relevant	82.86	.67 (.54-.79)*
Self-disclosure: Present	84.06	.71 (.54-.88)
Self-disclosure: Past year	88.41	.84 (.73-.96)
Self-disclosure: Perp is family	98.55	.98 (.95-1.02)
Self-disclosure: Perp is a friend	100.00	1 (1-1)
Self-disclosure: Perp is a partner	97.10	.97 (.92-1.01)
Self-disclosure: Perp is a man	88.41	.82 (.68-.95)
Self-disclosure: Perp is a woman	100.00	1 (1-1)
Self-disclosure: Perp is other	94.20	.93 (.85-1)
Self-disclosure: Rape	86.96	.74 (.58-.9)
Self-disclosure: Assault	100.00	1 (1-1)
Self-disclosure: Violate	100.00	1 (1-1)
Self-disclosure: Touched	98.55	.99 (.96-1.01)
Self-disclosure: Forced	98.55	.99 (.96-1.01)
Self-disclosure: Harassment	100.00	1 (1-1)
Self-disclosure: Catcall	100.00	1 (1-1)
Self-disclosure: Molest	97.10	.96 (.91-1.02)
Self-disclosure: Coerce	97.10	.97 (.93-1.01)
Self-disclosure: Online	95.65	.95 (.9-1.01)
Self-disclosure: Other	85.51	.83 (.72-.94)
Self-disclosure: Victim	92.75	.97 (.93-1.01)
Self-disclosure: Survivor	NC	.97 (.93-1.01)
Self-disclosure: Told others	92.75	.92 (.84-.99)
Depression	89.86	.85 (.74-.97)
Anxiety	91.30	.89 (.8-.98)
PTSD	NC	1 (1-1)
Suicide	95.65	.94 (.88-1.01)
Other mental health symptom	91.30	.88 (.78-.98)
Negative self-talk	92.75	.9 (.81-.99)
RM: No means Yes	100.00	1 (1-1)
RM: Women are asking for it	97.10	.97 (.93-1.01)
RM: Boys are naturally aggressive	100.00	1 (1-1)
RM: Alcohol leads to aggression	100.00	1 (1-1)
RM: Boys do not know better	97.10	.97 (.93-1.01)
RM: Conviction would ruin a life	100.00	1 (1-1)
RM: Women are lying	95.65	.95 (.9-1.01)
RM: Men cannot be victims	95.65	.95 (.9-1.01)
RM: Committed by strangers	95.65	.95 (.9-1.01)
RM: Survivors can prevent assault	95.65	.95 (.9-1.01)
RM: Will have good memory	98.55	.99 (.96-1.01)
RM: Other	86.96	.84 (.73-.95)
Dispels rape myth	73.91	.64 (.47-.82)

Providing resources	97.10	.97 (.93-1.01)
Help-seeking	95.65	.94 (.88-1.01)
Information seeking	95.65	.95 (.88-1.01)
Overall tone: Positive	95.65	.95 (.89-1.01)
Overall tone: Negative	84.06	.70 (.53-.87)
Overall tone: Neutral	91.30	.87 (.76-.98)
Overall tone: Mixed	86.96	.84 (.73-.95)

95% confidence interval
*n cases = 140, otherwise n=69
NC = not calculable due to missing data
RM = Rape myth
